# Educational Robotics to Foster and Assess Social Relations in Students' Groups

**DOI:** 10.3389/frobt.2020.00078

**Published:** 2020-06-26

**Authors:** Michela Ponticorvo, Franco Rubinacci, Davide Marocco, Federica Truglio, Orazio Miglino

**Affiliations:** ^1^Natural and Artificial Cognition Lab, Department of Humanistic Studies, University of Naples “Federico II”, Naples, Italy; ^2^Institute of Cognitive Science and Technology, National Research Council, Rome, Italy

**Keywords:** educational robotics, sociometric tools, social networks, assessment, students' groups, coding

## Abstract

Robotics has gained, in recent years, a significant role in educational processes that take place in formal, non-formal, and informal contexts, mainly in the subjects related to STEM (science, technology, engineering, and mathematics). Indeed, educational robotics (ER) can be fruitfully applied also to soft skills, as it allows promoting social links between students, if it is proposed as a group activity. Working in a group to solve a problem or to accomplish a task in the robotics field allows fostering new relations and overcoming the constraints of the established links associated to the school context. Together with this aspect, ER offers an environment where it is possible to assess group dynamics by means of sociometric tools. In this paper, we will describe an example of how ER can be used to foster and assess social relations in students' group. In particular, we report a study that compares: (1) a laboratory with robots, (2) a laboratory with Scratch for coding, and (3) a control group. This study involved Italian students attending middle school. As the focus of this experiment was to study relations in students' group, we used the sociometric tools proposed by Moreno. Results show that involving students in a robotics lab can effectively foster relations between students and, jointly with sociometric tools, can be employed to portrait group dynamics in a synthetic and manageable way.

## Introduction

During the last decades, different activities have found their own space along with curricular ones in schools. Between these, educational robotics (ER) can be an effective teaching and learning tool (Miglino et al., 1999) as it allows for transferring knowledge such as mathematics, computer science, and physics (Lindh and Holgersson, 2007; Williams et al., 2007; Nugent et al., 2009) and allows one to train skills, including thinking skills and problem solving approaches (Hussain et al., 2006; Sullivan, 2008; Mikropoulos and Bellou, 2013; Atmatzidou and Demetriadis, 2016; Gabriele et al., 2017).

An interesting review dating back to 2012 by Benitti (Benitti, 2012) reports that the use of robots in school has positive outcomes for teaching concepts that are connected to STEAM areas (STEM plus arts), as it can have an impact on education in the fields of science, technology, and mathematics along the educational process starting with preschool up to higher education including university (Javidi and Sheybani, 2010; Alimisis, 2013; Chung et al., 2014; Eguchi, 2014a).

Along the years, ER has been widely introduced in school activities and has consolidated its presence, especially in classrooms of high schools.

ER implies an integrated approach to complement different areas and fields, enhancing interest, and curiosity in scientific issues (Arís and Orcos, 2019).

For today's society, mastering technology is fundamental and ER can be used to introduce technology and promote other skills. In fact, in parallel with STEAM-related issues, ER allows to promote skills like initiative, autonomy, teamwork, and creativity (Sica et al., 2019a), the so-called 21st century skills (Eguchi, 2014b), complex, and evolutionary systems management (Miglino et al., 2004; Whittier and Robinson, 2007; Rubinacci et al., 2017a), together with social skills and communication (Owens et al., 2008).

A relevant study by Kandlhofer and Steinbauer (2016) shows that ER leads to a better achievement in social skills and self-esteem in students that results in increased motivation (Bazylev et al., 2014), which is a pivotal element in enhancing learning.

On the educational science side, ER is based on the constructionist approach, where the students are at the center of the learning challenge because they are active agents who can determine their learning processes (Piaget, 1974; Papert, 1980; Papert and Harel, 1991).

This means that, during ER activities, learners build their own pathways to understand the world around them; they discover, they use information to creatively get more knowledge, and they participate actively in the educational challenges, guided by teachers (Sica et al., 2019b).

Moving from the individual to the group level, it is interesting to underline that most of ER activities must be run in groups, thus promoting collaborative work and collaborative learning (Denis and Hubert, 2001). Collaborative learning in ER has been examined by a certain number of studies, showing how it can contribute to foster social ties in groups of students at different ages. The very recent study by Gonnot et al. (2019) analyzes the use of social robots in a context of collaborative learning, investigating how adding a social dimension to robot can improve learning. Some other studies were devoted to understand if social robots could affect the collaboration between children at play (Strohkorb et al., 2016) and to propose a framework for robots as mediator tools (Mitnik et al., 2008).

Robots can be the core element of an educational framework for collaborative learning, if they are conceived as components of Internet-of-Things (Plauska and Damaševičius, 2014), and, thanks to their features that promote collaborative learning, they can be used adopting a constructivist approach, as said before, which is highly motivating for children and adolescents.

The study by Atmatzidou and Demetriadis (2012) deepens the reflection on the pedagogical approaches for ER in the school context, which is a high-impacting issue. They explore different collaboration scripts used as a guide in students' group work during the ER activity.

## Robotics and Group Dynamics

Summarizing what literature taught, ER can be useful to promote the following: knowledge related to STEAM and skills such as computational thinking, problem solving, complex systems management, and collaborative learning, “inside the students,” which means that the focus is on the personal side.

In the present study, we propose to change the focus to what happens “between the students” who are involved in ER activities, which means that we concentrate on the social side.

We believe that ER can be used to foster positive and collaborative relations between students and, at the same time, provide a context to assess the changing networks in the classroom (Rubinacci et al., 2017b; Truglio et al., 2018a). In particular, these recent studies proposed by the authors of the present paper indicate how ER can be exploited to favor positive ties and connections between students.

Now we make a step forward to verify this claim and to show that the use of sociometric tools in the context of ER can picture the classroom environment in critical moments that affect students' career and classroom climate (Truglio et al., 2018b). A low social inclusion at school can have a dramatic effect on relevant phenomena including school dropout (Frostad et al., 2015; Ricard and Pelletier, 2016), and the sociometric framework offers sensitive tools to observe micro and macro dynamics elicited by ER activities.

This happens because ER allows one to establish a bridge between students, who become interdependent as they are required to reach a shared goal (Burbaite et al., 2013; Kamga et al., 2016), to coordinate themselves, to learn to divide tasks in subtasks, and to complete them, taking into account other group members (in terms of opinions, ideas, skills, and abilities). As a consequence, also those students who are not well-included in the class have the opportunity to be involved in group activity and to improve relationships with other students.

In this paper, we would like to show how ER is indeed an adequate and useful framework to assess social relations and support positive connections among students in the peer group. In particular, our research hypothesis is that ER can be more effective in promoting positive ties and connections between students if compared with other activities. At the same time, our goal is to verify if the use of sociometric tools offers a valid framework to evaluate these ties. To address these issues, we have worked on a 2-month project (from September to November 2017). The trial took place in Naples and its surroundings, an area in Southern Italy, which is highly affected by school dropout resulting in threats at the social level (O'Higgins et al., 2007). In the section Robotics to Foster and Assess Social Relations in Students' Groups, we will describe this project in more detail including the results we obtained, and in the section Discussion and Conclusions, we will discuss these results and their implications.

## Robotics to Foster and Assess Social Relations in Students' Groups

In this section, we describe the study we have run in a secondary school in Italy. The proposed research project aims to assess whether the ER laboratory, through group activities, is an effective method to assess, and promote social relationships within a peer group in the class. To test our research hypothesis, we considered three groups and two activities: the ER laboratory and the coding laboratory with Scratch. The third group performed individual activities that were not intended to stimulate interactions between students. It was thus possible to picture the group dynamics at the beginning of the school year and the effect of the different activities on them.

### Materials and Methods

#### Participants

The study involved 70 participants attending the first-year of middle school (“Scuole medie” in the Italian school system), aged between 10 and 11 years. Thirty-eight participants were females and 32 males; their mean age was 10.48 years.

We decided to focus on first-year students as there are weak ties between them, especially at the beginning of the school year.

For school needs, each group was randomly assigned to a condition of the experimental design. From discussion with school referents, we were assured that classes were composed so as to be homogenous in terms of grades from primary school, gender balance, and social skills.

In more details:

1. Group 1, formed by 23 students, carried out the ER laboratory.
2. Group 2, formed by 24 students, performed the coding laboratory with Scratch.
3. Group 3, composed of 23 pupils, was not involved in any group activity.

Group 3 was the control group and allowed to obtain the baseline to compare group activities about the effects on social relations, as the time lapse between the start and the end of group activities may anyway have an effect on links and relationships between peers.

#### The Tools for Group Activities: Lego Mindstorms NXT and Scratch

The robotics technology we used in the present project was Lego Mindstorms NXT (Klassner and Anderson, 2003). This robotics kit includes both a hardware side and a software side (NXT-G).

In the coding lab, we used Scratch (Maloney et al., 2010): it is a programming language that is freely available and is commonly used to approach children, kids, and teen students to coding as it offers the opportunity to create multimedia and interactive games simply and intuitively with images, music, and sounds. Together with coding, related to computational thinking, this software helps students to develop their skills related to creativity, systematic reasoning, and problem solving.

#### Sociometric Test

To assess social relations, the sociometric test of Moreno was administered to the students of the three groups, before and after the laboratory activities. The sociometric test allows one to effectively investigate interpersonal relationships inside the peer group and to highlight the status of the group components in terms of inclusion. Indeed, sociometry is a methodology that was proposed by Jacob L. Moreno in order to study the structure and interactions of people within a group (Moreno, 1941, 1951), and it has been employed in many different contexts including family therapy and educational contexts. If we consider educational contexts, the sociometric methodology can be useful to examine situations where there are conflicts among students, isolated subjects, lack of cooperation in working groups, etc. In the present project, it was the selected tool to picture and study the links between peers in the three groups involved in the activities.

The sociometric test proposed to the students concerned the criterion that is called affective-relational perspective. This perspective is related to the emotional aspect of a relationship and reflects students' affinities.

The criterion is operationalized in two sentences, which allow to highlight preferences and, conversely, rejections toward members of the group. These sentences ask to indicate the classmates who the responding participants would (or would not) want as roommates during a school trip.

Then the first step is to report data in a double-entry table named sociomatrix. In this table, on the axes of abscissas and ordinates, there are the names of the group members: horizontally we report the expressed choices (or rejections) and vertically the received choices (or rejections). The choices are indicated with “1” and the rejections with “−1.”

Let us consider an example of a very small group of children, composed by A, B, C, and D.

We will then have a square matrix with four elements on the axes of abscissas and ordinates. If A chooses B, we will put a +1 at the intersection between A (horizontally) and B (vertically). If C rejects D, we will put a −1 at the intersection between C (horizontally), and D (vertically).

**Figure 1:**
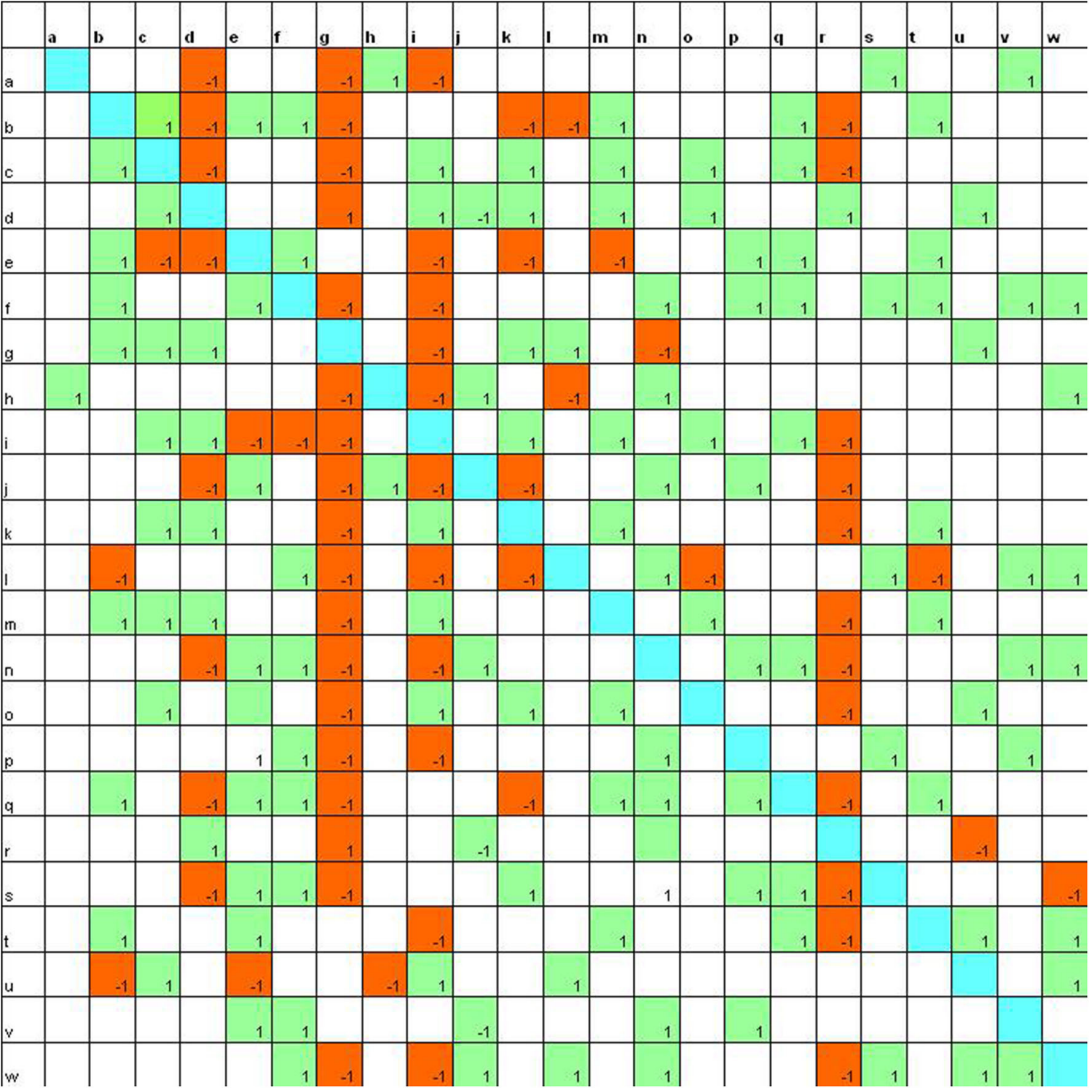
Sociomatrix built on the *Educational Robotics group* for the affective criterion at the beginning of the scholastic year.

**Figure 2:**
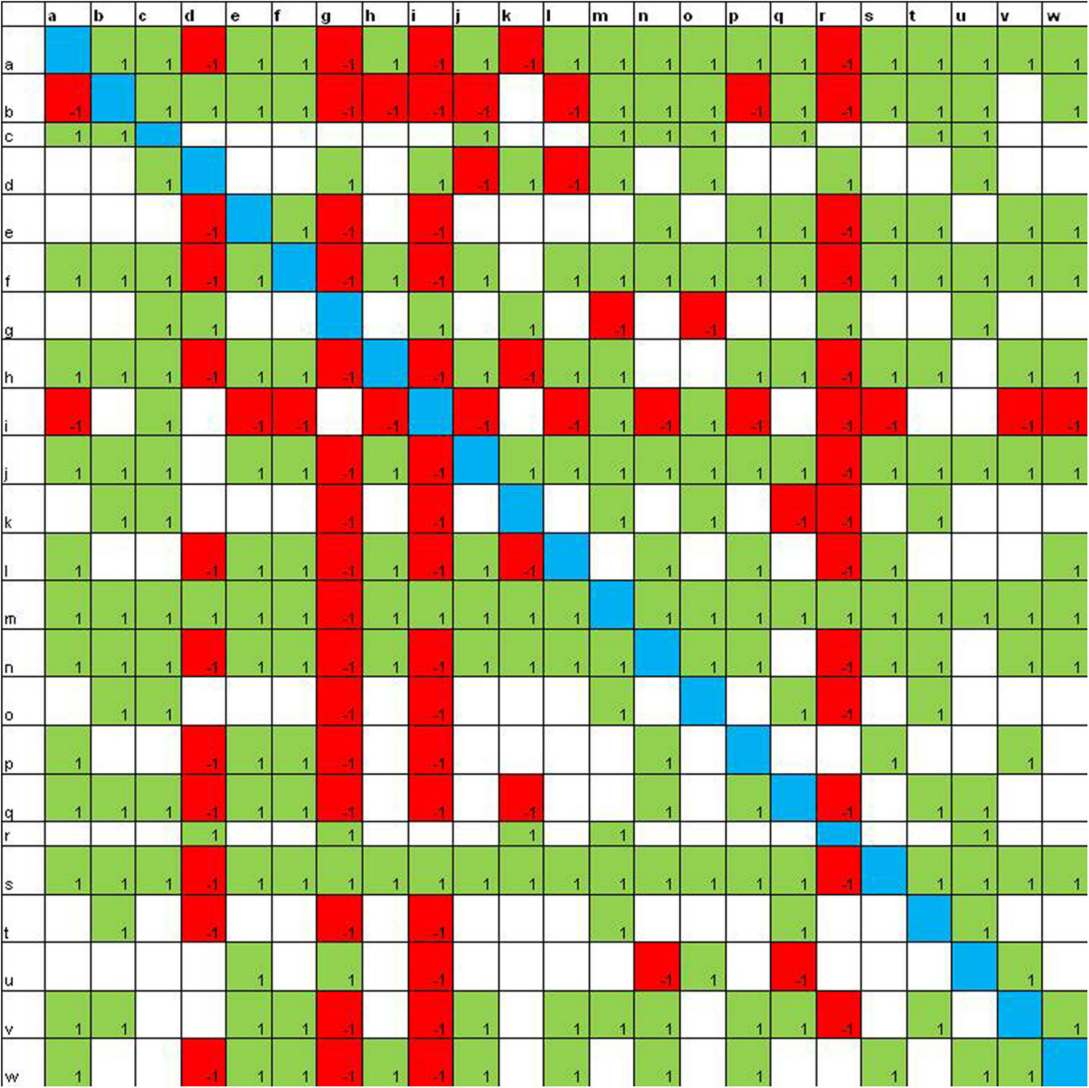
Sociomatrix built on the *Educational Robotics group* for the affective criterion at the end of the robotics laboratory.

**Figure 3:**
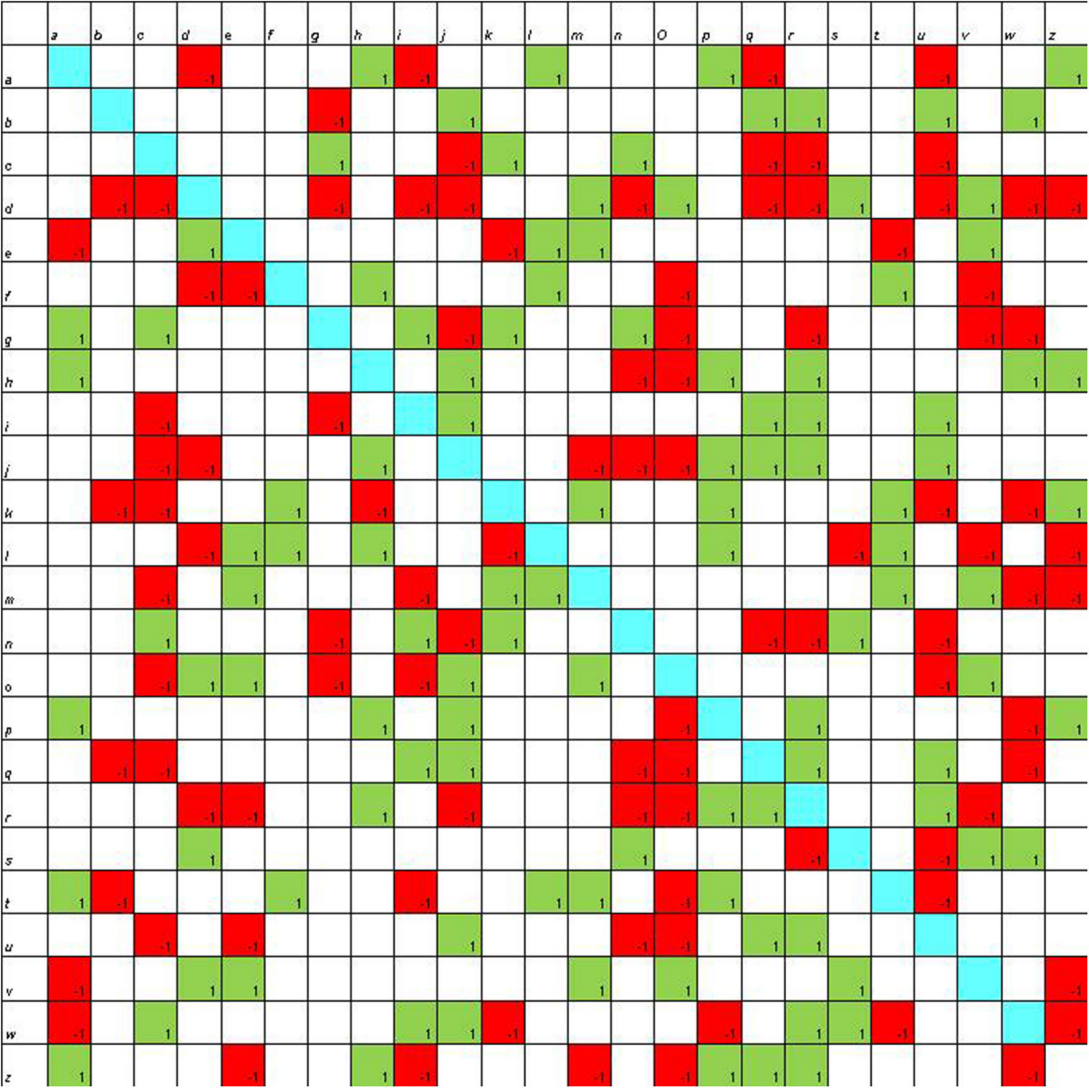
Sociomatrix built on the *coding with Scratch group* for the affective criterion at the beginning of the scholastic year.

**Figure 4:**
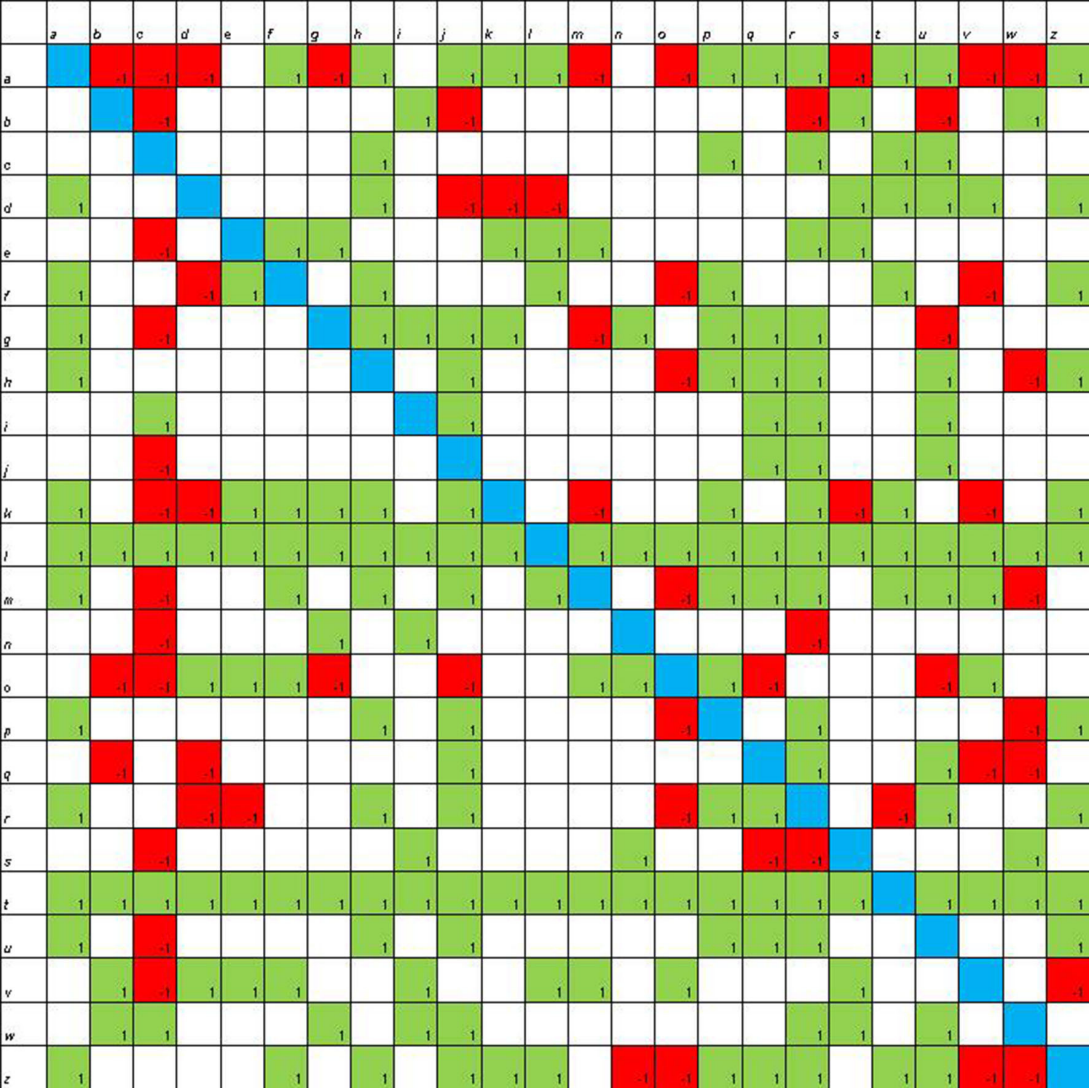
Sociomatrix built on the *coding with Scratch group* for the affective criterion at the end of laboratory activities.

**Figure 5:**
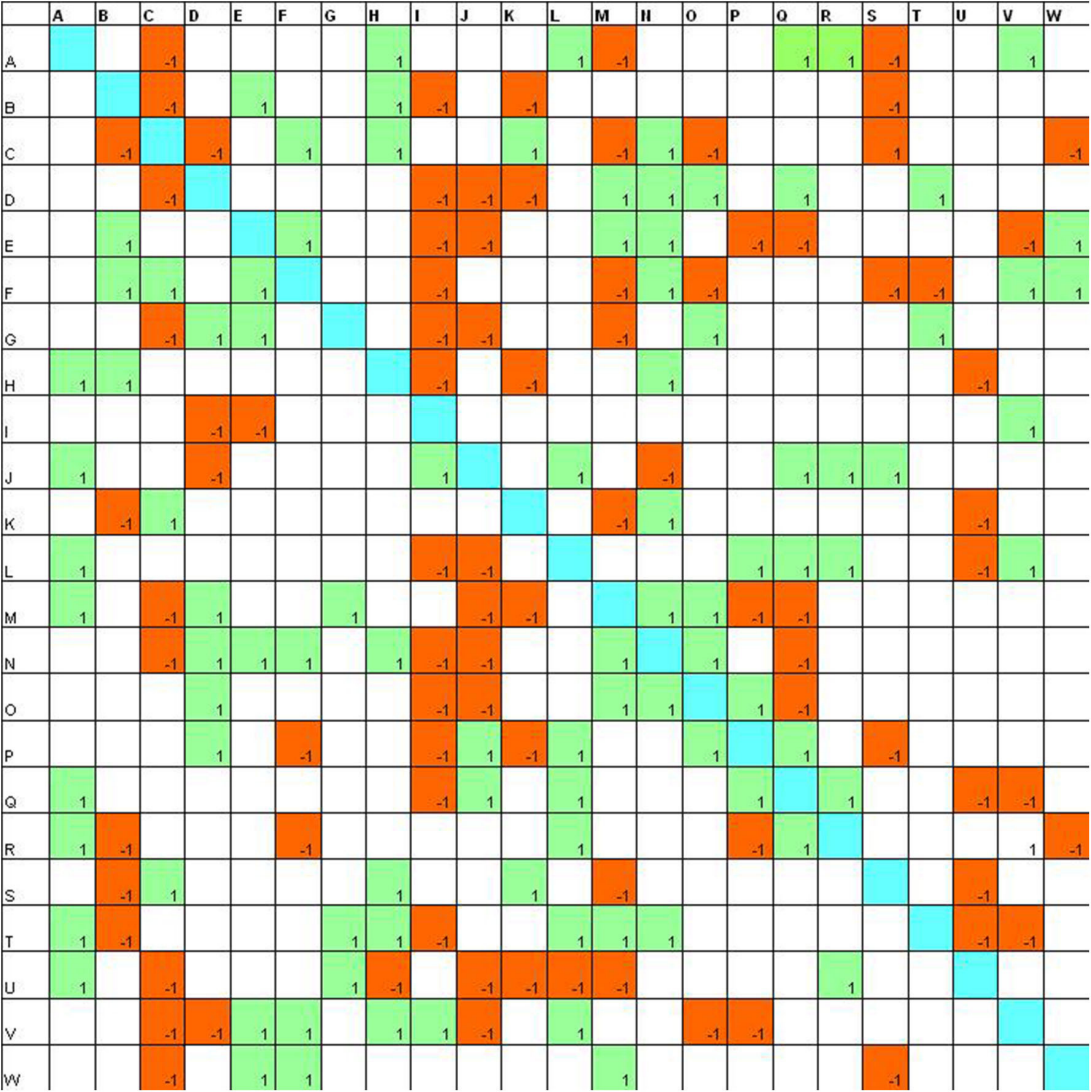
Sociomatrix built on the control group for the affective criterion at the beginning of the scholastic year.

**Figure 6:**
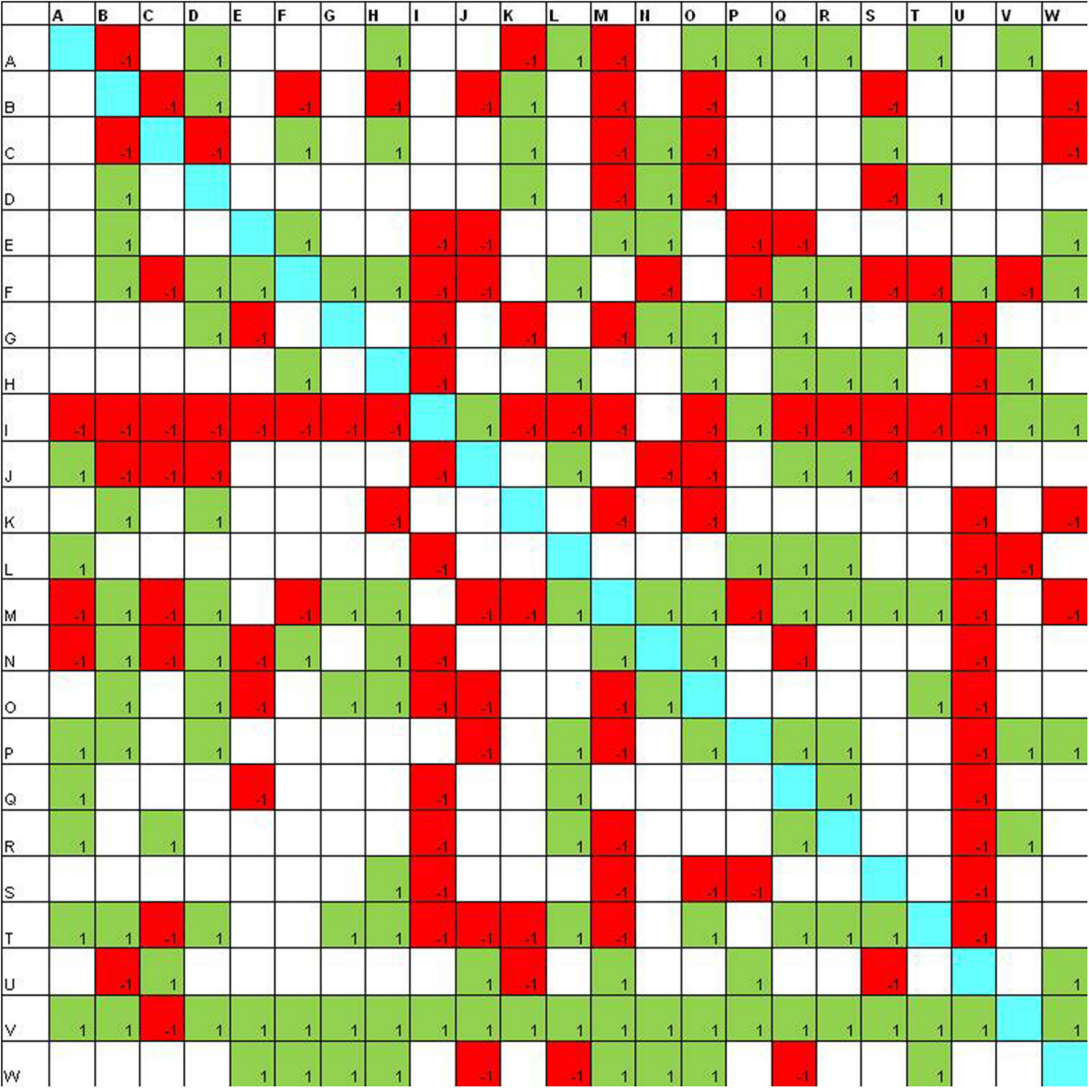
Sociomatrix built on the control group for the affective criterion at the end of laboratory activities.

**Figure 7:**
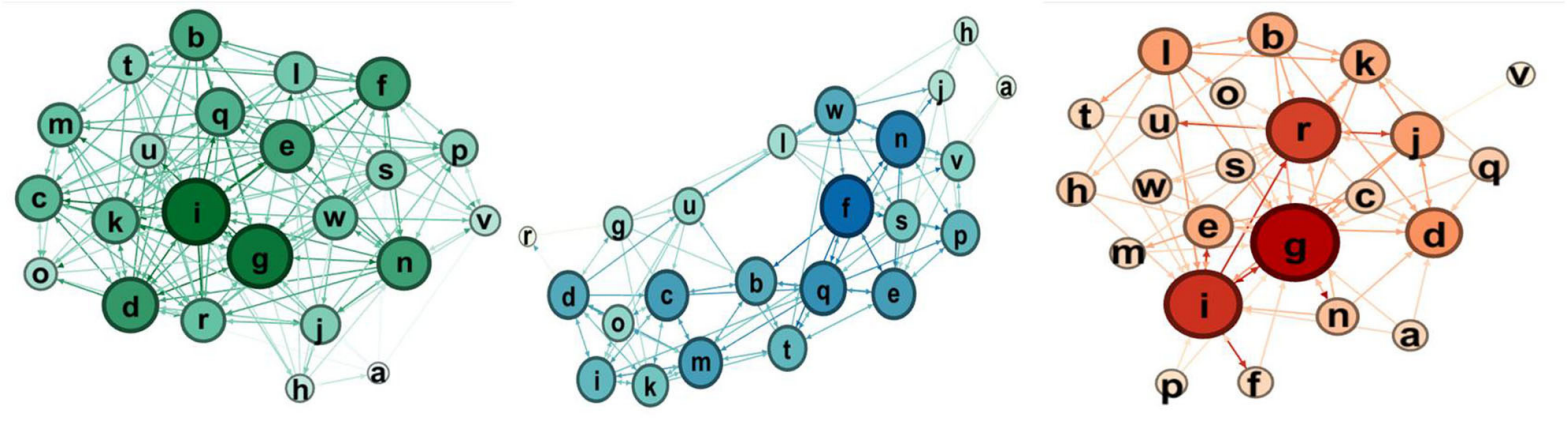
Sociograms representation of *Educational Robotics group* at the pretest. In green the total one, in blue the selection one, and in red the rejection one.

**Figure 8:**
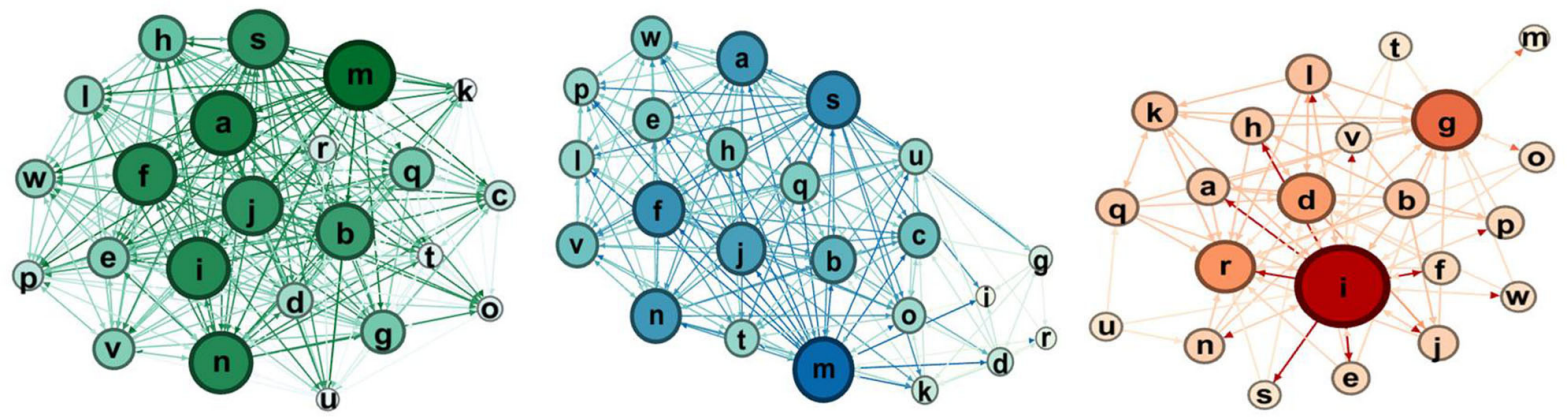
Sociograms representation of *Educational Robotics group* at the post-test. In green the total one, in blue the selection one, and in red the rejection one.

**Figure 9:**
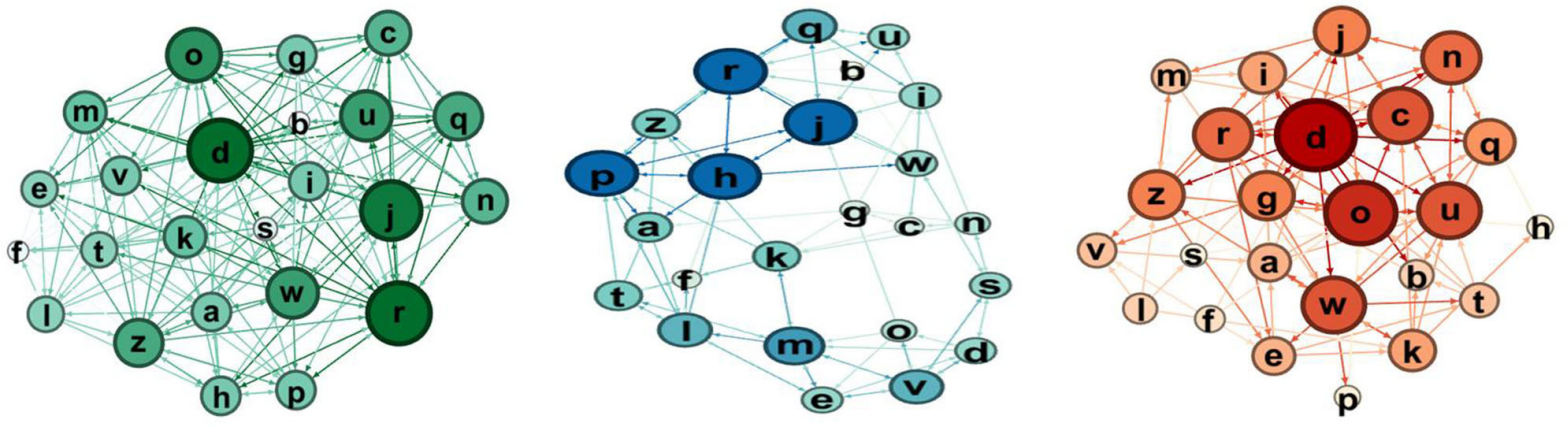
Sociograms representation of the coding group at the pretest. In green the total one, in blue the selection one, and in red the rejection one.

**Figure 10:**
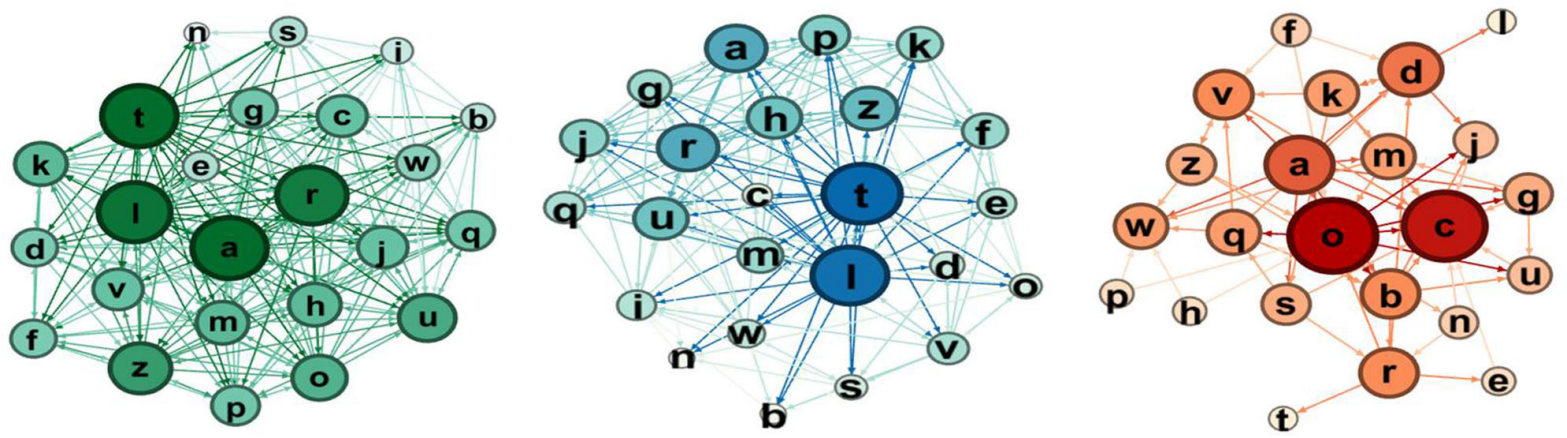
Sociograms representation of the coding group at the post-test. In green the total one, in blue the selection one, and in red the rejection one.

**Figure 11:**
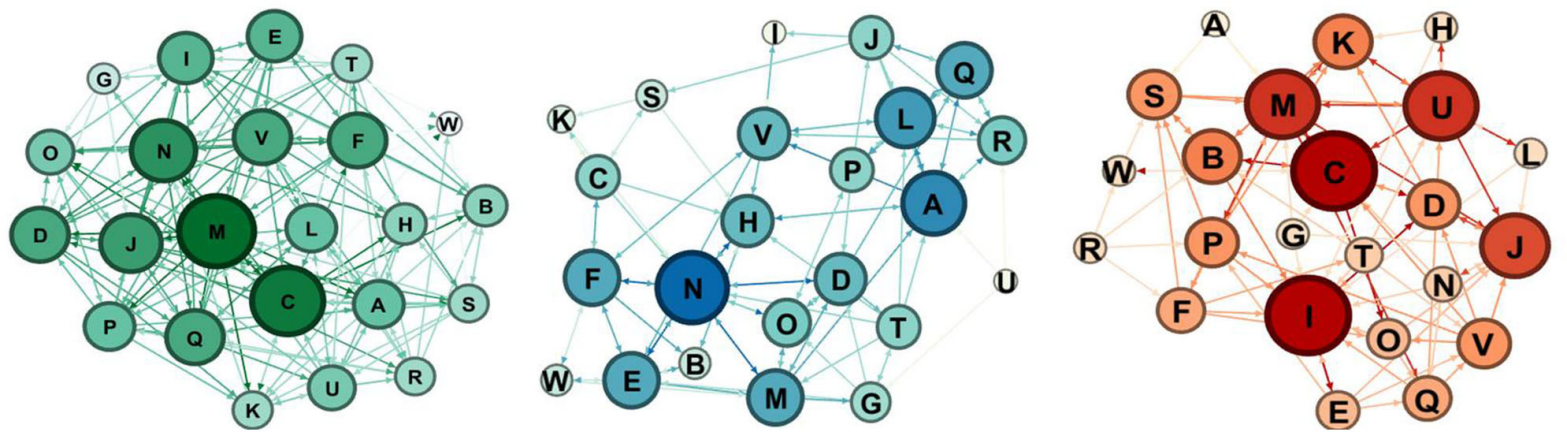
Sociograms representation of the control group at the pretest. In green the total one, in blue the selection one, and in red the rejection one.

**Figure 12:**
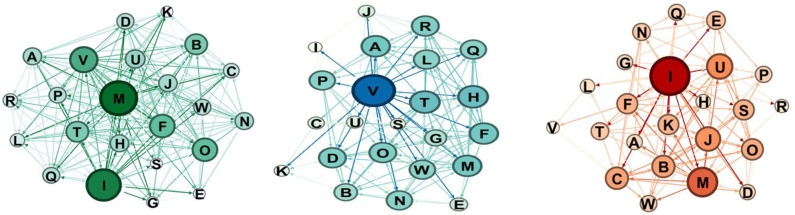
Sociograms representation of the control group at the post-test. In green the total one, in blue the selection one, and in red the rejection one.

These sociometric data can be represented in the graphical form called sociogram too. It is a network graph with nodes and lines. Nodes represent the students, the components of a group, whereas lines are the links, the relations (different kinds of lines distinguish choices and rejections). Furthermore, each line has one or two arrows showing the direction of the relationship and if it is unidirectional or bidirectional. In the sociogram, A, B, C, and D will be the nodes, pictured as circles, and they will be connected by lines with different graphical features, corresponding to a different kind of relation (see the Figures 1–12 in the section Discussion and Conclusions).

From the sociogram and the sociomatrix, it is possible to delineate the following: the total choices and rejections that each member of the group has received; the degree of reciprocity of choices and rejections; and the difference between ignored, rejected, isolated, and popular subjects.

The popular subjects are those who have received a large number of choices, so they are those who have greater influence and greater power within the group. The rejected subjects, on the other hand, are those who have received a large number of rejections. Finally, the isolated subjects are those who have received very low number of choices. This last category includes:

1. subjects who are ignored by the group, but who prove to be open and available to others by expressing their choices and
2. subjects who are ignored and tend to self-isolate by expressing neither choices nor rejections.

Along with the sociogram and the sociomatrix, it is possible to use statistical techniques on the indexes that are derived from the sociometric tools. The sociometric tool provides a rich amount of data on group interaction and dynamics.

#### Procedure

As hinted at previously, the three conditions to verify the effects of different activities on interpersonal relations in the peer group are the lab with robotics activities and the lab with Scratch about coding, together with the control group. The groups have been randomly assigned to one of the three experimental conditions (ER lab, coding lab with Scratch, and no group activities).

The sociometric test was proposed to the participants (the students belonging to the three experimental groups) in two moments, on September 25 (i.e., before the beginning of lab activities: pretest) and on November 29 (i.e., at the end of lab activities: post-test).

The activities covered 6 weeks, with a meeting for a week and each lasting 1 or 2 h (for a total of 10 h). To carry out the activities, students were divided in subgroups that were composed by different students at each meeting. In the next subsection, laboratory activities are described in more detail.

#### The Laboratory Activities

During the laboratory activities, which were scheduled as 6 weekly meetings lasting 1 or 2 h, participants followed different pathways.

In summary, in the robotics lab, the following activities were carried out: realization of posters dealing with technology, robot building and programming, and building of road itineraries representing the environment where the robot moved.

For the coding lab with Scratch, the students were involved in the following: realization of posters regarding the topic of technology, creation of a *sprite* (an element of Scratch programming environment, which can be conceived as an agent; see Ponticorvo et al., 2017), creation of the *stage* (the place where sprites interact), coding of *sprite* behavior in a spatial labyrinth, and building of multimedia road itineraries representing the environment where the *sprite* moved. These activities were conceived in order to make comparable the tasks with the students attending the robotics lab and the coding lab.

What is different is that in the robotics lab, participants used tangible materials, so as to build the robot and to realize road itineraries, whereas those in the coding lab have carried out their activities exclusively with software, then in a digital environment.

Previous work conducted by our research group indicates that this element can be relevant in promoting different cognitive and social processes (Di Fuccio et al., 2015; Ferrara et al., 2016).

In more detail, the ER lab's schedule was the following: during the first meeting, by a frontal and interactive lesson, the researcher talked about technology and introduced the definition of a robot as an artifact with a sensory-motor system. At the end of this first meeting, participants are divided in five subgroups to build autonomously and collaboratively a poster about technology and robots. During the second meeting, students were divided again in five subgroups, different from the previous ones. Every subgroup built a robot using the tools described above. Students had to collaborate and work in groups to reach a common goal.

In the third meeting, the software to program the robot was introduced and students used it to implement the robot control system. Participants were again divided in subgroups and worked together to build their strategy for the robots. In the fourth and fifth meetings, they built the street pathways for the robot taking inspiration from their own city and elaborating them in a creative way. The sixth meeting was devoted to writing the code and transferring it in the robot to follow the street pathway, always working in subgroups.

The coding laboratory with *Scratch* was structured in a very similar way: in the first meeting by a frontal and interactive lesson, the researcher talked about technology, and introduced the software *Scratch* for programming. At the end of the meeting, students were divided into five subgroups to build autonomously and collaboratively a poster about technology and *Scratch*. In the second meeting, run in the computer classroom, *Scratch* was introduced in its basic functionalities. Later participants were divided in subgroups and had realized together some elements in *Scratch*.

In the third meeting, how to program the elements in *Scratch* had been shown and then they were divided in subgroups to decide their strategy to program to follow a spatial labyrinth.

During the fourth and fifth meetings, students, divided in subgroups, implemented multimedia, taking inspiration from their city. In the last meeting, a street pathway had been implemented and new subgroups had been formed to write the code and the sequences to follow the multimedia street pathway.

### Results

In this section, we report sociograms and sociomatrices for each condition at the beginning and at the end of the intervention, we compare the indexes for the three conditions, and then we confront the number of selections and rejections at the beginning and at the end of the project using *t*-test.

#### Sociogram and Sociomatrix Analysis

Sociograms and sociomatrices were built on the three groups both for pretest and post-test. Group 1 carried out the ER laboratory, Group 2 performed the coding laboratory with Scratch, and Group 3 was the control group. Here we report sociograms and sociomatrices about the affective criterion.

In the sociomatrices, to delineate the choices of the members of the group, the number 1 was used inside a green box, and to indicate the rejection, the −1 was used in a red box. Furthermore, the total choices and the total rejections (both expressed and received) were recorded for every student in the peer group.

At the beginning of the school year (Figure 1), it is possible to observe that there are six students who are able to attract a good number of choices (8 and 9, the highest values) and two students who receive more than 10 rejections.

After the laboratory activities (Figure 2), it is evident that peers make much more selections and more students receive a high number of choices (16 students receive 10 or more choices). Also the rejections increase and four students receive more than 10 rejections.

In the group that was involved in the coding activities, at the beginning of the school year, there are three students who attract 8–9 choices and one student who receives 10 rejections (Figure 3).

Also in this case, the number of choices increases (Figure 4): seven students receive 10 or more selections and only one student receives more than 10 rejections.

In the control group, at the first assessment (Figure 5), there are two participants who receive 8–9 selections and one participant who collects more than 10 rejections.

At the end of the project (Figure 6), the number of choices increases and three participants receive more than 10 rejections.

The sociomatrix represents the basis for other analysis and allows one to have a relevant number of information in a synthetic way.

Starting from the sociomatrices, we built the sociograms and calculated various indexes, as described by Garcia-Magarino et al. (2019), with the software Gephi, an open-source software package for analysis and visualization of social networks (Bastian et al., 2009).

Here we report the sociograms for the three experimental groups at the pretest and post-test, considering the total one, i.e., the one that considers both selections and rejections and then, separately, the selections and the rejections.

The qualitative comparison of the sociograms at these two moments shows some interesting dynamics. In the robotics group (Figures 7, 8), the network becomes more connected: in particular, the selection one shows much more links. Considering the node in the rejections groups, at post-test, there is only one node that receives a high number of rejections.

For the coding group (Figures 9, 10), we observe that there are much more choices, especially selections as rejections link decreases.

If we consider rejects, in the control condition, rejects increase significantly, whereas in the robotics condition, the number of rejects remains essentially the same.

For the control group (Figures 11, 12), the comparison between the pretest and the post-test indicates that the group has more links, as we expected for the time lapse, but it is interesting to notice that the rejection links increase.

In Table 1, the analysis run with the Gephi software is reported at the pretest and the post-test. The average corresponds to the ratio between connections (edges) and the number of participants (nodes). Here, the Social Intensity, Cohesion, Dissociation, and Coherence indexes are also reported (Garcia-Magarino et al., 2019).

**Table 1:** Indexes for the experimental groups at pretest and at post-test obtained with the Gephi software.

	**Nodes**		**Edges**		**Average**		**Index**		**Coherence**	
	**Pre**	**Post**	**Pre**	**Post**	**Pre**	**Post**	**Pre**	**Post**	**Pre**	**Post**
**Robotics Group**										
Total (Social Intensity index)	23	23	196	323	8.52	14	0.387	0.638	0.56	0.62
Selection (Cohesion index)	23	23	126	239	5.48	10.39	0.25	0.472	0.67	0.65
Rejection (Dissociation index)	23	22	70	84	3	3.8	0.138	0.182	0.2	0.33
**Coding Group**										
Total (Social Intensity index)	24	24	206	255	8.59	10.6	0.373	0.462	0.53	0.42
Selection (Cohesion index)	24	24	107	193	4.46	8	0.194	0.35	0.64	0.48
Rejection (Dissociation index)	24	24	99	162	4.125	2.7	0.179	0.123	0.30	0.16
**Control Group**										
Total (Social Intensity index)	23	23	184	5 262	8	11.4	0.364	0.518	0.55	0.53
Selection (Cohesion index)	23	23	98	146	4.3	6.3	0.194	0.289	0.65	0.41
Rejection (Dissociation index)	23	23	86	116	3.7	5	0.17	0.229	0.33	0.34

*Average is the ratio between connections (edges) and the participants (nodes). The column Index reports the Social Intensity, Cohesion, Dissociation indexes; the column Coherence indicates the coherence of the social network*.

*Social Intensity Index* measures the percentage of relations (reciprocal or not) on the number of theoretically possible combinations. It indicates how the students are connected, either positively, or negatively. Usually, a high value of the index means that students know each other well.

*Cohesion Index* is the ratio between reciprocal relations and possible relations. Cohesion is useful to understand if students rely on the others in the group. It is the level of reciprocal acceptation between students and can highlight popular students.

*Dissociation Index* represents the opposite of previous metrics because it is centered on the ratio between reciprocal rejects and the number of possible combinations. This index shows the average ratio of reciprocal rejects and if there are unpopular students.

*Coherence* refers to the ratio between reciprocal selections and selections received by other students. In other words, it represents the reciprocity in students' selection. It is useful to highlight if students tend to have reciprocal relations.

These indexes vary between 0 and 1.

Table 1 summarizes the indexes for the experimental group at the pretest and the post-test.

Coherently with what we have observed by the sociograms, the indexes get better between the pretest and the post-test. There is a notable increase in Social Intensity and Selection indexes and a low increase in Rejection for the robotics lab.

These analyses indicate that the robotics lab can be effective in promoting dynamics that can lead to a modification of the status of each participant at a personal level and of the group as a dynamic entity.

In the coding laboratory, there is a little increase in Social Intensity and Selection indexes and a little decrease in Rejection: this indicates that the network has changed slightly. In the control group, all indexes increase a little, as expected because of the interaction related to school.

In the three experimental conditions, the indexes show an increase between pretest and post-test selections.

To better understand the effects produced by the robotics lab in comparison with the coding activity, we run the statistical analyses whose results are reported in the next section.

#### Statistical Analysis on Choices and Rejections

In this section, we report the analysis on the number of choices and rejections in the robotics lab, the coding lab, and the control group: in particular, we analyzed the difference between selections and rejections at the beginning and at the end of the project. Is there a difference considering the beginning of activities and the end? Results on this research question are reported in Table 2.

**Table 2:** Comparison between pretest and post-test about received selections and rejects in the three experimental conditions (*p* < 0.05 are marked with an asterisk).

	**Selections**		**Rejects**	
	***t*-test**	***p*-value**	***t*-test**	***p*-value**
Robotics	7.71507274	≃0*	1.834425	0.079561
Coding	6.87784727	≃0*	3.279852	0.003286*
Control	3.73671073	0.001079202	2.998793	0.006408*

Is robotics more effective than the other conditions to foster relations in the peer group? To answer this question, we have compared the three conditions in two moments: the pretest and the post-test running a one-way ANOVA with the software SPSS®.

At the pretest, the ANOVA revealed no significant differences between the three conditions: F_(2, 67)_ = 1.803; *p* = 0.173 for selections and F_(2, 67)_ = 0.574; *p* = 0.566 for rejections.

On the contrary, at the post-test, the difference is significant if we consider the selections: F_(2, 67)_ = 7.569; *p* = 0.001 (for selections). The *post-hoc* comparisons (Bonferroni method) indicate that a statistically detectable difference emerges between the robotics condition and the control group: average difference = 4.043; *p* = 0.001.

## Discussion and Conclusions

ER is nowadays a frequent appointment in curricular pathways; the experiment we have described and the related data indicate a notable change in the interpersonal relations within the group that attended the robotics lab in the direction of their improvement. This change emerges in the comparison with the control group and the coding lab. This result can be motivated by the shift of the learning perspectives, which becomes more active, and consequently by the different way students interact with each other. Indeed, according to the constructivist approach, this kind of activities offers the students the possibility to establish relations with their peers in a different way in order to understand their psychological affinities. To solve the robotics tasks, the participants must act in an interdependent way, whereas the majority of curricular activities are individual. Allowing to move from individual to group activity forces to build an interdependent relation: the students who are not well-included in the peer group have a new chance to be an active part in solving the tasks thus improving relationships with other students. The present study has indeed some limitations; for example, it was run on already established groups (classes), so it was not possible to vary the group composition. Moreover, the groups were followed along a relatively short period of time, and it would be interesting to verify if the positive changes were stable over time.

From these results, it is possible to deduce that labs and related activity can be an effective methodology to promote and support new and satisfying relations between students. The data reported in the Results section indicate that there is an increase in selections in the ER condition, which is higher than the other conditions, thus showing that the ER activities can have some specific features that are functional to improve the relations between peers, which is, in turn, a protective factor to prevent dropout.

Some issues remain still open: in the group that was involved in the robotics lab, a small number of participants remain rejected. Does it depend on the individual participant or from the group organization? And if it depends on the individual, which are the psychological variables that are relevant?

Future research will be devoted to address this question, along with the comparison with the educational robotic lab with different activities that foresee interactions with tangibles (such as laboratories of craft, art, workshop on music, etc.). These new experiments will investigate if social relations can be enhanced specifically by running a lab or if this improvement is comparable to the effects of other group activity involving manipulation.

This first project was followed by a wider experience under the Codinc project (Coding for inclusion) in the period January–May 2019. In this European-funded project, ER, together with sociometric tools, has been the context to assess peer relations and has become the core of Codinc methodology, as it offers the opportunity to portray interpersonal relationship in a situation that is different from the common interactions of the peers at school.

## Data Availability Statement

The datasets generated for this study are available on request to the corresponding author.

## Ethics Statement

Ethical review and approval was not required for the study on human participants in accordance with the local legislation and institutional requirements. Written informed consent to participate in this study was provided by the participants' legal guardian/next of kin.

## Author Contributions

MP, FR, and OM conceived the original idea and planned the experiments. FT carried out the experiment. MP, FR, FT, and DM run the statistical analyses. MP took the lead in writing the manuscript with the support of FR. FT and OM supervised the findings of this work. All authors discussed the results and contributed to the final manuscript.

## Conflict of Interest

The authors declare that the research was conducted in the absence of any commercial or financial relationships that could be construed as a potential conflict of interest.
